# Infectious Progeny of 2009 A (H1N1) Influenza Virus Replicated in and Released from Human Neutrophils

**DOI:** 10.1038/srep17809

**Published:** 2015-12-07

**Authors:** Zhang Zhang, Tao Huang, Feiyuan Yu, Xingmu Liu, Conghui Zhao, Xueling Chen, David J. Kelvin, Jiang Gu

**Affiliations:** 1 Department of Pathology and Provincial Key Laboratory of Infectious Diseases and Immunopathology, Collaborative and Creative Center, Shantou University Medical College, Shantou, Guangdong, 515041, China; 2 Department of Pathology, Beijing University Health Science Center, Beijing, 100083, China; 3 Division of Experimental Therapeutics, Toronto General Research Institute, University Health Network, Toronto, Ontario, Canada; 4 Department of Immunology, Faculty of Medicine, University of Toronto, Toronto, Ontario, Canada; 5 Universita’ degli Studi di Sassari, Sezione di Microbiologia Sperimentale e Clinica, Dipartimento di Scienze Biomediche, Viale San Pietro 43/b, 07100 Sassari, Italia; 6 International Institute of Infection and Immunity, Shantou University Medical College, Shantou, Guangdong, China; 7 Institute of Medical Science, Faculty of Medicine, University of Toronto, Toronto, Ontario, Canada; 8 Translational Medicine Center, Second Affiliated Hospital, Shantou University Medical College, Shantou, China

## Abstract

Various reports have indicated that a number of viruses could infect neutrophils, but the multiplication of viruses in neutrophils was abortive. Based on our previous finding that avian influenza viral RNA and proteins were present in the nucleus of infected human neutrophils *in vivo*, we investigated the possibility of 2009 A (H1N1) influenza viral synthesis in infected neutrophils and possible release of infectious progeny from host cells. In this study we found that human neutrophils *in vitro* without detectable level of sialic acid expression could be infected by this virus strain. We also show that the infected neutrophils can not only synthesize 2009 A (H1N1) viral mRNA and proteins, but also produce infectious progeny. These findings suggest that infectious progeny of 2009 A (H1N1) influenza virus could be replicated in and released from human neutrophils with possible clinical implications.

Various *in vitro* and *in vivo* studies have shown that neutrophils can be infected with a number of viruses included influenza virus. For example, the migratory and phagocytic activities of murine neutrophils can be altered by an *in vitro* interaction with cytomegalovirus^1^, and activated neutrophils had vacuoles containing varicella-zoster virions and extended cytoplasmic projections toward virions^2^. A number of viruses attached and penetrated into neutrophils have been observed by Blackmon and Ginsberg^3^. The infection of influenza virus and its effect on neutrophil function such as suppressing endocytosis, accelerating apoptosis and inducing type I interferon signaling pathways have been extensively studied^4^,^5^,^6^,^7^,^8^,^9^. We also observed neutrophils infection by H5N1 virus *in vivo*^10^.

The successful replication of virus depends on host cells to provide functional organelles such as ribosome and endoplasmic reticulum to synthesize viral mRNA and proteins. Neutrophils, as mature polymorphonuclear leukocytes (PMNs), generally thought to have no significant biosynthetic capacity, synthesizing little if any RNA and protein^11^. It was reported that neutrophils have only few endoplasmic reticulum, mitochondria and ribosome^12^. Traditionally, it was believed that influenza viral infection of neutrophils is abortive, and infectious progeny are not produced. There was no detectable level of viral progeny in the supernatant of cytomegalovirus, varicella-zoster virus or even the A/WSN/33 (H1N1) strain of influenza virus infected neutrophils *in vitro*^1^,^2^,^13^.

However, Jack and Fearon reported that human peripheral blood neutrophils can selectively synthesize mRNA and proteins^14^. Several studies have also shown that the rate of RNA and protein synthesis increased upon neutrophil stimulation^8^,^11^,^15^,^16^,^17^. In our own study, we found that H5N1 viral proteins and nucleotide sequences were present in both the cytoplasm and the nucleus of infected neutrophils^10^. It has been shown that influenza virus synthesizes its messenger RNA in the nucleus of infected cells^18^,^19^. Based on the presence of human avian influenza virus RNA and proteins in the nuclei of infected human neutrophils, we speculate that human neutrophils may be infected by influenza virus, and serve as host cells for virus replication and progeny production.

To verify our hypothesis, we first examined the expression of sialic acid, the primary receptors for influenza virus, on neutrophils^20^. We further examined the infection, replication and progeny release of 2009 A (H1N1) virus in human neutrophils *in vitro*. Unexpectedly, we found that human neutrophils *in vitro* without detectable level of sialic acid expression could be infected by the virus. We also found that the infected neutrophils can not only synthesize 2009 A (H1N1) viral mRNA and proteins, but also produce infectious progeny. To our knowledge, this is the first observation of mature virions produced by neutrophils.

## Results

### The separated human neutrophils are of good quality

**Figure 1:**
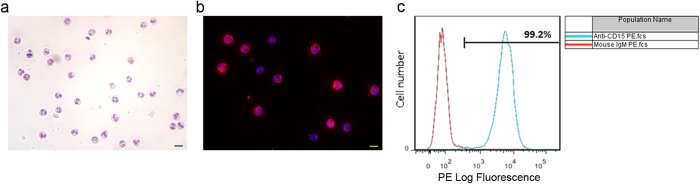
The separated human neutrophils are of good quality. (**a**) The polymorphonuclear characteristic of human primary neutrophils was determined with Giemsa staining. (**b**) The expression of CD15 on separated cells was analyzed with immunofluorescence. The primary antibody was mouse anti-CD15, the secondry antibody was Alexa Fluor 555 conjugated goat anti-mouse (red), and the nuclei were stained with DAPI (blue). (**c**) Flow cytometry was performed to depict the purity of neutrophils with PE-conjugated anti-CD15 antibody (blue curve). PE-conjugated mouse IgM was used as the isotype control (red curve). Scale bar, 20 μm.

The quality of separated human neutrophils is essential for functional tests. The morphology of separated cells was highly consistent with specific polymorphonuclear characteristics of neutrophils which was identified with Giemsa staining (Fig. 1a). To further confirm the identity of neutrophils, immunofluorescence staining and flow cytometry were performed with mouse anti-CD15 (a marker of neutrophils) monoclonal antibody^10^. Figure 1b shows that the CD15^+^ cells had the neutrophil characteristic morphology of lobulated nuclei. Flow cytometry found that the purity of neutrophils reached 99.2% (Fig. 1c). Therefore, the quality of neutrophils separated is adequate for carrying out functional assays.

### Influenza virus can enter neutrophils independent of sialic acid receptors

**Figure 2:**
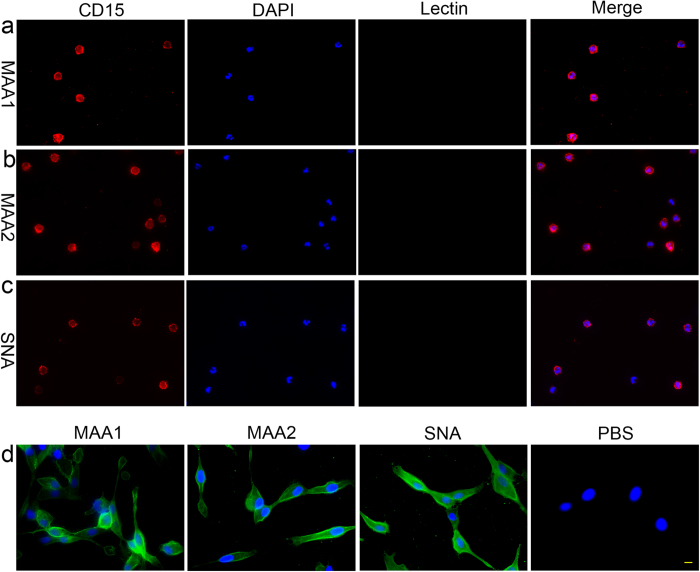
No sialic acid residue, which is the primary receptor for influenza virus, was detectable on human neutrophils *in vitro*. (**a–c**) The human primary neutrophils were incubated with biotinylated MAA I, MAA II or SNA, subsequently incubated with Dylight 488 labeled Streptavidin (green) and examined with immunofluorescence. No detectable level of sialic acid in α2–3 linkages (a&b) or sialic acid in α2–6 linkages (**c**) was found on neutrophils. The cells were stained with mouse anti-CD15 antibody (red) to identify the identity of neutrophils. (**d**) Lectin stain was also performed on MDCK cells as a positive control to ensure the reliability of the technology. Nuclei were stained with DAPI (blue). Scale bar, 20 μm.

The expression of sialic acid on neutrophils and MDCK cells was examined with fluorescence microscopy using MAA I, MAA II and SNA stainings. As shown in Fig. 2a-c, no detectable level of sialic acid in α2–3 linkages or in α2–6 linkages was found on neutrophils with MAA I, MAA II or SNA staining. Meanwhile, antibody to CD15 was used to identify neutrophils. As a positive control, MDCK cells showed a strong expression of both avian influenza receptors (α2,3-linked sialic acids) and human influenza receptors (α2,6-linked sialic acids) to ensure the reliability of the technology (Fig. 2d).

To further clarify whether the entry of influenza virus into neutrophils is independent of sialic acid receptors, neutrophils were preincubated with neuraminidase (NA) prior to infection with influenza virus. Fig. S1 shows that the percentage of neutrophils pretreated with NA had nucleoprotein positive cells (infected cells) in number not less than that of the group of neutrophils without NA treatment.

### 2009 A (H1N1) viral mRNA and protein can be synthesized in human neutrophils *in vitro*

To examine the proliferation of 2009 A (H1N1) influenza virus in human neutrophils *in vitro*, real time quantitative PCR, Western blot and immunofluorescence staining were performed.

**Figure 3:**
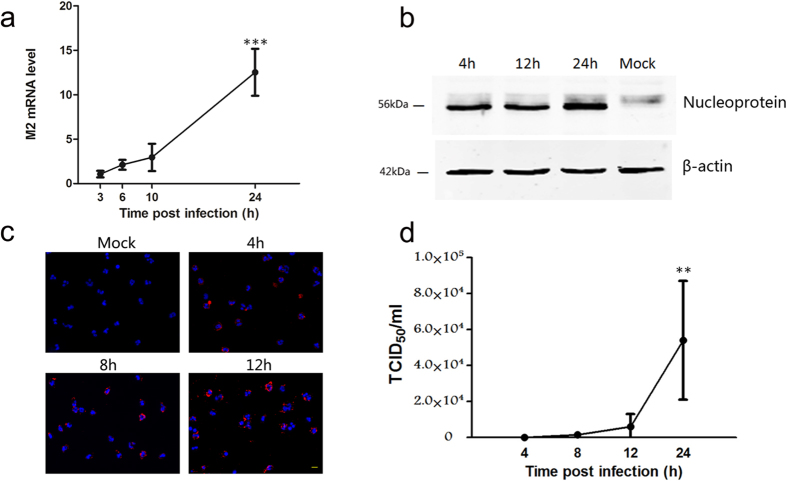
2009 A (H1N1) Influenza Virus replicated in and released from Human neutrophils *in vitro*. (**a**) Time course of 2009 A (H1N1) virus matrix 2 (M2) mRNA synthesis was analyzed with Real-time PCR. Data are shown as mean ± S.D. ***P < 0.001, indicate statistically highly significant differences of M2 mRNA expression at 24 h post infection comparing with its expression at 3 h, 6 h and 10 h post infection (P = 0.0001, 24 h vs 3 h post infection; P = 0.0002, 24 h vs 6 h post infection; P = 0.0008, 24 h vs 10 h post infection), n = 4. (**b**) The accumulation of virus Nucleoprotein at different time points post infection was examined with Western blot. (**c**) In situ analysis of 2009 A (H1N1) virus matrix 1 (M1) protein synthesis at different time points post infection with immunofluorescence. Scale bar, 20 μm. (**d**) The influenza virus titers of supernatants from infected neutrophils at different time points post infection were examined with the TCID50 assay. Data are shown as mean ± S.D. *P < 0.05, indicate statistically highly significant differences of virus titers at 24 h post infection comparing with virus titers at 4 h, 8 h and 12 h post infection (P = 0.0168, 24 h vs 4 h post infection; P = 0.0189, 24 h vs 8 h post infection; P = 0.029, 24 h vs 12 h post infection), n = 4.

With real time quantitative PCR, we analyzed the expression of influenza A virus matrix 2 (M2) mRNA isolated from virus-exposed neutrophils. The M2 gene production was encoded by the spliced mRNA, which was only present within the infected cells^9^. As shown in Fig. 3a, along with prolonged post-infection duration, the expression levels of the M2 gene gradually increased and became very strong at 24 h after infection.

With Western blot, we examined the expression of influenza A virus Nucleoprotein in virus-exposed neutrophils at 4 h, 12 h and 24 h post infection (Fig. 3b). Virus nucleoprotein displayed a constant level of protein accumulation with a little difference in protein accumulation levels observed at 4 h and 12 h post infection. After 24 h, protein expression levels increased significantly. The results were consistent with that of real time quantitative PCR.

With immunofluorescence, we examined the expression of influenza virus matrix 1 (M1) in cells at a series of time points post infection. As shown in Fig. 3c, along with prolonged post-infection duration, not only the number of infected cells constantly increased, but also the signal strength of positive cells apparently enhance.

### Infectious progeny virus was released from viral infected human neutrophils *in vitro*

The culture supernatant of 2009 A (H1N1) virus-infected neutrophils was examined with the TCID_50_ assay to assess the production of infectious progeny virus and viral replication kinetics. Mature viral particles were found to be released from infected neutrophils and increased persistently as the duration prolonged (Fig. 3d). The infection and replication kinetics of H1N1 virus in neutrophils obtained from different donors were consistent.

**Figure 4:**
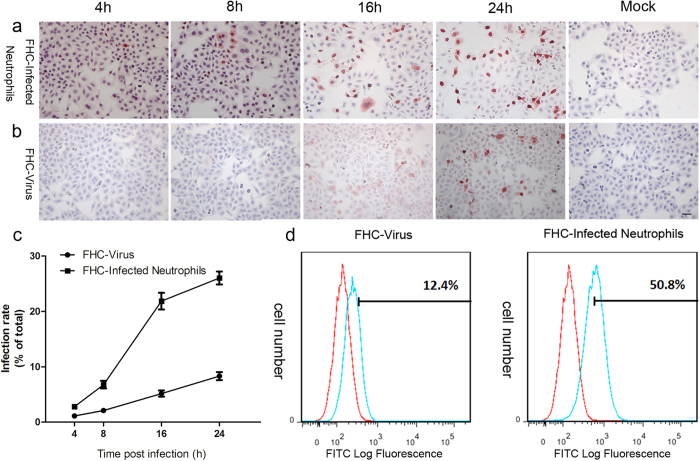
The infectivity of progeny virus released from neutrophils was examined with cell co-culture. (**a,b**) Immunocytochemistry shows time course of 2009 A (H1N1) virus Nucleoprotein expression in FHC cells co-cultured with infected neutrophils or incubated with parent 2009 A (H1N1) virus directly. Scale bar, 20 μm. (**c**) Statistical data of different cell infection rates between (**a,b**), n = 4. (**d**) Flow cytometry shows the comparison of different cell infection rates between FHC cells incubated with 2009 A (H1N1) virus directly and FHC cells co-cultured with infected neutrophils at 24 h post infection using FITC-conjugated mouse anti-Nucleoprotein antibody (blue curve), uninfected neutrophils were used as negative control (red curve).

The infectivity of progeny virus released from neutrophils was also examined with cell co-culture of a different cell type (FHC). As depicted in Fig. 4a, following designated duration of cell co-culture with infected neutrophils, influenza virus Nucleoprotein expression was detected with immunocytochemistry in FHC cells. The expression level of virul Nucleoprotein in the FHC cells at 4 h post infection was very low. With the increase of viral replication cycles, infected neutrophils gradually released more mature viral particles. The numbers of infected FHC cells were significantly increased at 8, 16 and 24 h post infection. The amounts of virus nucleoprotein positive signals were remarkably enhanced at 24 h (Fig. 4a). Compared with the control group (Fig. 4b), in which FHC cells were directly incubated with parent virus propagated in embryonated eggs, we found that the rate of infected FHC cells co-cultured with virus-infected neutrophils was much higher (Fig. 4c). At 24 h post infection, flow cytometry demonstrated that the control group (FHC cells directly incubated with parent virus) had an average positive signal expression rate of 12.4%, and the experimental group (FHC cells co-cultured with virus-infected neutrophils) had an average rate of 50.8% (Fig. 4d), represent an increase of reflecting an infection rate increase of over fourfold.

### The conditions of neutrophils before and after infection with 2009 A (H1N1) virus

**Figure 5:**
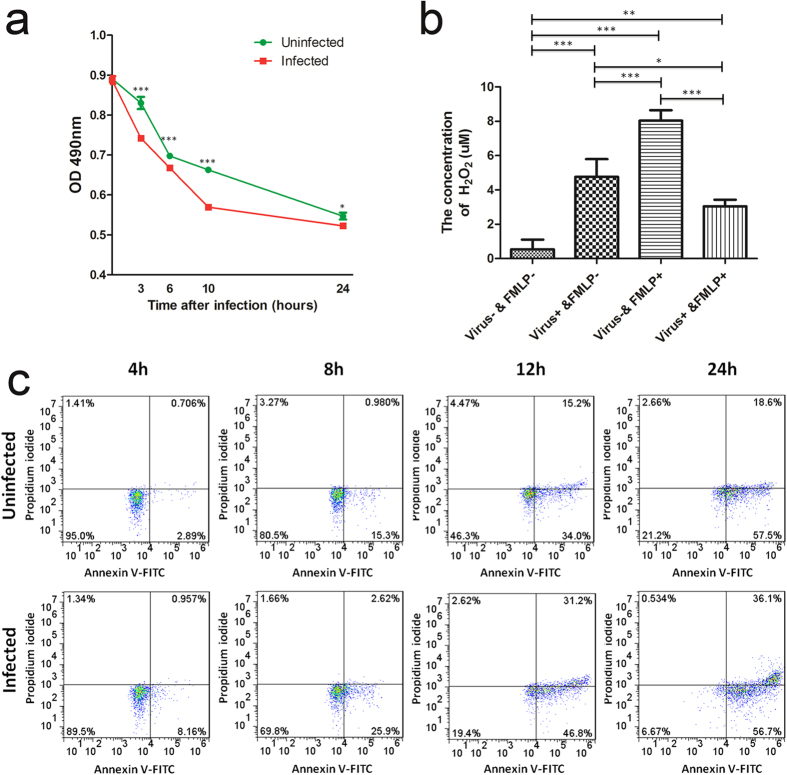
The conditions of neutrophils before and after infection with 2009 A (H1N1) virus. (**a**) The viability of infected neutrophils was significantly lower than that of the uninfected neutrophils examined with MTS assay. Data are shown as mean ± S.D. *P < 0.05, ***P < 0.001, n = 3. Differences between infected and uninfected samples were analysed with Student’s t-test. (**b**) Hydrogen Peroxide production of neutrophils was higher following treatment with virus for 30 min than the untreated controls (Virus+ & FMLP– vs Virus– & FMLP–); but it also depressed H_2_O_2_ production of neutrophils to mount respiratory burst responses to FMLP (Virus+ & FMLP+ vs Virus- & FMLP+). Data are shown as mean ± S.D. *P < 0.05, **P < 0.01, ***P < 0.001, n = 3. Differences among groups were analysed with One-way ANOVA. Comparisons between any two groups were performed with q-test (Newman-Keul’s test). (**c**) Influenza virus accelerated neutrophil apoptosis, which was proved with Annexin V-FITC/PI staining at different time points post infection.

In order to better understand the interaction of 2009 A (H1N1) influenza virus with neutrophils, the conditions of neutrophils before and after infection were examined. As shown in Fig. 5a, influenza virus significantly reduced the viability of neutrophils at all time points post infection. Meanwhile, neutrophil apoptosis was accelerated following infection with influenza virus (Fig. 5c). The respiratory burst responses of neutrophils before and after infection were also examined through monitoring the production of H_2_O_2_. As shown in Fig. 5b, influenza virus itself induced a respiratory burst in which H_2_O_2_ were production (Virus+ & FMLP– vs Virus– & FMLP–), but it also depressed the ability of neutrophils to mount respiratory burst responses to FMLP (Virus + & FMLP + vs Virus– & FMLP+).

## Discussion

Our previous research found that H5N1 viral proteins and nucleotide sequences were present in both the cytoplasm and the nucleus of infected neutrophils of H5N1 virus infected patients^10^. This prompted us to speculate the possibility of influenza virus multiplication in human neutrophils. A number of studies on the ability of viral replication in neutrophils were reported about 30 years ago, but none found any evidence of successful viral replication^1^,^2^,^13^. To our knowledge, this is the first report of infectious viral progeny production and release by neutrophils.

Neutrophils have little biosynthetic activities as only a scanty endoplasmic reticulum and little ribosomes or mitochondria were present in these cells^11^,^12^. However, several studies observed that RNA and protein syntheses by neutrophils were increased after stimulation^8^,^11^,^15^,^16^,^17^ indicating that neutrophils still possess the abitity to supply sufficient biosynthetic components necessary for viral replication. The observed replication of 2009 H1N1 strain in neutrophils might indicate that this strain might be able to stimulate neutrophils to synthesize viral components and assemble infectious viral progeny. The reason for A/Nanchang/8002/2009 H1N1 but not A/WSN/33 (H1N1)^13^ successfully replicate in neutrophils remains to be investigated.

Cell surface sialic acid residues were known as primary receptors for influenza viruses^20^, but the distribution of sialic acid on neutrophils has not been carefully examined. We performed lectin staining with MAA and SNA on neutrophils and no detectible level of sialic acid, nor α-2,3-linked or α-2,6-linked was found, while positive controls ensured the reliability of our technical protocal. Two probabilities exist: Low level sialic acid residues might exist on neutrophil surface that might be below the detecting sensibility of lectin staining, or 2009 A (H1N1) virus strain can enter neutrophils without the classic salic acid receptors. As shown in Fig. S1, there was no difference in the percentage of infected cells between groups of neutrophils with and without NA pretreatment. This result indicates that even low level of sialic acid residues exist on neutrophil surface, sialic acid-dependent entry may not the main route for influenza virus to infect neutrophils. There is likely that other sialic acid receptors independent route(s) of entry might exit for influenza virus to infect neutrophils.

Cumulating evidence indicates that salic acid was not the sole gateway for influenza virus infection. It was reported that NWS-Mvi and parent influenza viruses could infect desialylated cells^21^. A/Memphis/1/71 (H3N2) influenza virus was found to bind to galactosyl ceramide (GalCer: Galβ1 → 1′Cer), as well as sulphatide, in virus overlay assays^22^. Macropinocytosis is yet another pathway for viruses to enter neutrophils without binding to specific receptors^23^. Phagocytosis is a major strategy for neutrophils to defend against invading pathogens, and this is also a possible pathway for viruses to enter neutrophils^24^. Inconsistence between the distributions of classic avian influenza virus receptor and infected cells in H5N1 virus infected patients was also reported^25^,^26^. The way and means of viral entry into neutrophils remain a subject of farther investigation.

One of the major cause of morbidity and mortality during influenza virus epidemics is bacterial superinfection^27^. Phagocyte dysfunction induced by influenza virus is one likely contributor to the development of bacterial superinfection, in addition to damage to respiratory epithelium^28^,^29^. Neutrophils and monocyte/macrophages infiltrate at the site of virus infection, and participate in the early inflammatory response. Both cell types exhibit depressed function *in vivo* during influenza virus infection^30^. Substantial evidences have been documented about the effect of influenza viruses on neutrophil function. Accelerated apoptosis^5^ and defects in chemotactic, oxidative and bacterial killing functions^28^ of neutrophils have been established in influenza virus infection. In addition, influenza virus itself can induce activation of neutrophils to generate a respiratory burst response^31^, but impair the ability of neutrophil respiratory burst respond to other stimuli^32^. In this study, we examined the conditions of neutrophils before and after infection by 2009 A (H1N1) strain virus. In accordance with other reports, 2009 A (H1N1) strain virus reduced cell viability, accelerated cell apoptosis, activated neutrophils itself and deactivated the ability of the cells to respond to FMLP (Fig. 5). Neutrophil dysfunction might be resulted from previous activation by influenza virus, inducing cell deactivation and viability reduction.

The pandemic 2009 A (H1N1) disease showed clinical symptoms similar to seasonal influenza, including fever, cough, sore throat, headache, myalgias, and arthralgias^33^. However, it also displayed symptoms that were not commonly seen in seasonal influenza including gastrointestinal symptoms such as diarrhea and vomiting, or neurological complications such as seizures, and encephalopathy^34^,^35^. Autopsies of deceased patients observed erythrophagocytosis and phagocytosis of inflammatory cells in various organs^36^ similar to those observed in patients infected with highly pathogenic avian influenza virus (HPAIV H5N1).

Our previous studies demonstrated multiple organ infections outside the lungs in H5N1 infected patients^25^. The pathologic findings of these autopsies included hemophagocytic activities in the spleen, liver, lymph node, and bone marrow^37^,^38^,^39^,^40^, white pulp atrophy with depletion of lymphocytes in the spleen^25^,^38^,^39^; apoptotic lymphocytes in the spleen and the intestine^40^; acute tubular necrosis^25^,^39^; activated Kupffer cells, cholestasis, fatty liver^25^,^39^,^40^, and edema of the brain^25^,^39^. What’s more, the H5N1 virus penetrated the placental barrier and infected the fetus^25^. Pandemic 2009 A (H1N1) virus was found to replicate in the intestine of the ferrets besides respiratory tract, a sign of multiple organ infection^41^. Previous reports demonstrated that infection by 2009 H1N1 virus was not confined to the upper respiratory tract but also involved the lungs and other organs, most importantly immune cells^36^,^42^. Many fatal patients had lymphopenia and elevated creatinine kinase (CK)^43^,^44^,^45^. Lymphocytic disruption caused an acute immunodeficiency that resulted in acute progressive respiratory syndrome including lower respiratory tract disease, respiratory failure, and ARDS with refractory hypoxaemia^43^,^44^. Based on our current findings that neutrophils can be infected by 2009 A (H1N1) and permit active viral replication, we hypothesize that infected immune cells including neutrophils may act as vehicles, transporting the virus to other organs and causing extra-pulmonary dissemination and multiple organ infection. The extent of immune system infection may be a determinant factor for the disease.

In conclusion, the present study shows that human neutrophils without detectable level of sialic acid expression on their surface can be infected by 2009 A (H1N1) influenza virus. Such virus can be replicated in and released by neutrophils and may play a role in the pathology and multiple organ infection with significant clinical implications.

## Methods

### Ethics Statement

All experiments involving human participants and chicken embryos were approved by the Ethical Committee of Shantou University Medical College. All participants have provided written informed consent. All methods were carried out in according to relevant national and international guidelines.

### Separation of human neutrophils

Healthy adult Chinese donors’ heparinized whole blood (n = 10) was mixed with an equal volume of 3% dextran T-500 (Pharmacosmos, Holbaek, Denmark) in 0.9% NaCl and incubated 20 min at room temperature. Aspirated leukocyte-rich plasma and centrifuged 10 min at 1000 rpm, 5 °C. Discarded supernatant and resuspended cell pellet in a volume of 0.9% NaCl equal to the starting volume of blood. Layered 10 ml Ficoll-Hypaque solution (Solarbio, Beijing, China) beneath the cell suspension which ≤40 ml, and centrifuged 40 min at 1400 rpm, 20 °C with no brake. Aspirated the top layer as well as the Ficoll-Hypaque layer, leaving the neutrophil/RBC pellet, and removed residual RBC with red blood cell lysis buffer (TBDScience, Tianjin, China). After washing with Hanks buffered saline solution (Solarbio, Beijing, China), the cells were resuspended and cultured in IMDM (HyClone/Thermo Fisher Scientific Inc., Waltham, MA, USA) supplemented with 10% fetal bovine serum (FBS, HyClone), 100 IU/ml of penicillin G and 100 μg/ml streptomycin (Invitrogen, Carlsbad, CA, USA) in a cell incubator (5% CO_2_ at 37 °C)^46^. The morphological characteristics and purity of neutrophils were examined with Giemsa staining, immunofluorescence staining and flow cytometry as described below.

### Giemsa staining

To examine the morphological characteristics of purify cells, Giemsa staining (Solarbio, Beijing, China) was performed according to the manufacturer’s protocol. In brief, purified cells were fixed with methyl alcohol for 3 min, and then incubated with Giemsa stain for 20 min at room temperature. After washing with water and drying in the air, the slides were examined and photographed with a light microscopy (Leica, Wetzlar, Germany).

### Lectin staining

The distribution of sialic acid in neutrophils and Madin-Darby canine-(MDCK) cells was examined with lectin staining. Human neutrophils and MDCK cells were fixed with 4% paraformaldehyde, and incubated with 5% BSA for 30 min at room temperature. The cells were incubated with biotinylated Maackia amurensis agglutinin (MAA I, MAA II, Vector laboratories, Burlingame, CA, USA), which binds to the avian influenza receptor Siaα2-3Galβ1-3GalNAc, or biotinylated Sambucus nigra agglutinin (SNA, Vector laboratories, Burlingame, CA, USA), which links to the human influenza receptor Siaα2-6Galβ1-3GalNAc^47^,^48^,^49^,^50^, and mouse anti-CD15 antibody (neutrophils marker) for neutrophils only at 4 °C overnight. After washing with PBS, they were incubated with Dylight 488 labeled Streptavidin (Vector laboratories, Burlingame, CA, USA) and Alexa Fluor 555 conjugated goat anti-mouse antibody (Life/Thermo Fisher Scientific Inc., Waltham, MA, USA) for neutrophils only for 1 h at room temperature. After a final wash, DAPI (Vector laboratories, Burlingame, CA, USA) was utilized to counterstain cell nuclei. A fluorescence microscope (Carl Zeiss, Oberkochen, Germany) was used for evaluation and photographing.

### Neuraminidase treatment of neutrophils

After separation, 1 × 10^6^ neutrophils were incubated with 0.128 U/ml of α2-3,6,8,9 Neuraminidase A (New England Biolabs, Inc., Ipswich, MA, USA) at 37 °C for 1 hour with constant mixing followed by washed three times with PBS and resuspended in influenza virus growth medium for infection.

### Virus preparation and infection

Influenza virus A/Nanchang/8002/2009 H1N1strain was propagated in embryonated eggs, and the allantoic fluid was harvested. Virus titers were determined by tissue culture infection dose (TCID50) assay via infection of MDCK cells. Virus was stored at 70 °C until use^51^.

Infections were performed as previously described^51^. Briefly, we thawed virus stock in cool water which was then used at a infection (m.o.i.) of 1 plaque forming units (p.f.u.)/cell incubated with neutrophils or MDCK cells for 1 h at 33 °C in a CO_2_ incubator. After viral adsorption, unadsorbed viruses were washed away with warm HBSS (Solarbio, Beijing, China) and then cells were maintained in growth medium at 37 °C in a CO_2_ incubator before analysis.

### Real time PCR and relative gene expression analysis

Total cellular RNA was extracted at the designed hours post infection with Trizol reagent (Takara, Dalian, China). cDNA was synthesized from mRNA with Random primers and MultiScribe^TM^ reverse transcriptase (Applied Biosystems/Thermo Fisher Scientific Inc., Waltham, MA, USA). The mRNA levels of target gene were quantified by Real-time quantitative PCR analysis with an Applied Biosystems 7500 Real-Time PCR System. Amplification of β-actin was performed in parallel as an internal control. The primers used in this study were: Influenza A virus matrix 2 (M2) (accession number: CY089611.1): sense, 5′-GAGGTCGAAACGCCT-3′, antisense, 5′-CTGTTCCTTTCGATATTCTTCCC-3′^9^, β-actin (accession number: NM_001101.3): sense, 5′-AGCGAGCATCCCCCAAAGTT-3′, antisense, 5′- GGGCACGAAGGCTCATCATT-3′. The relative expression of M2 mRNA was determined with the method of 2^−△△Ct^^52^.

### Immunofluorescence staining

Immunofluorescence staining was performed according to a protocol described previously with slightly modification^53^. At 4 h, 8 h, and 12 h post infection, neutrophils attached on glass slides coated with fibronectin (Sigma, St. Louis, MO, USA) in advance were fixed with 4% paraformaldehyde, permeabilized and blocked with 0.2%Triton X-100, 5%BSA. The slides were incubated overnight with mouse anti-CD15 monoclonal antibody (eBioscience, San Diego, CA, USA) or mouse anti-Influenza A virus matrix 1 (M1) antibody (Abcam, Cambridge, MA, USA) as the primary antibody. After washing in PBS, slides were incubated with Alexa Fluor 555 conjugated goat anti-mouse antibody (Life/Thermo Fisher Scientific Inc., Waltham, MA, USA). VECTASHIELD mounting medium DAPI (Vector laboratories, Burlingame, CA, USA) was applied to nuclear staining. The slides were examined and photographed with a fluorescence microscope (Carl Zeiss, Oberkochen, Germany).

### Western blot

Western blot was performed essentially the same as previously described^54^. Briefly, cells were lysed in RIPA buffer (Cell Signaling Technology, Boston, MA, USA) containing protease and phosphatase inhibitors (Roche, Penzberg, Germany). The concentrations of protein in the lysates were determined with Pierce BCA Protein Assay Kit (Thermo Fisher Scientific Inc., Waltham, MA, USA) according to the manufacturer’s protocol. After electrophoresis on 10% SDS-PAGE Bio-Rad minigels, the aliquots proteins were transferred to nitrocellulose filter membrane (Whatman/GE Healthcare life sciences, Marlborough, MA, USA). The blots were blocked for 1 hour at room temperature in 5% skim milk in TBST, and incubated overnight with rabbit anti-Influenza A Virus Nucleoprotein antibody (GeneTex, Irvine, CA, USA) and mouse anti-β actin monoclonal antibody (ZSGB-BIO, Beijing, China) at a dilution of 1:10,00 as primary antibodies. IRDye 680-conjugated goat polyclonal anti-rabbit IgG (H+L) and IRDye 800-conjugated goat polyclonal anti-mouse IgG (H+L) (both from LiCor, Lincoln, NE, USA) were used as secondary antibodies at a dilution of 1:10,000. The blot was examined and photographed with a Odyssey imaging systems (LiCor, Lincoln, NE, USA).

### Flow cytometry

PE-conjugated anti-CD15 monoclonal antibody (eBioscience, San Diego, CA, USA) and FITC-conjugated anti-Influenza A Virus Nucleoprotein monoclonal antibody (Abcam, Cambridge, MA, USA) were used for flow cytometry. For surface marker staining, neutrophils were blocked with 5% BSA to avoid nonspecific reaction and incubated with PE-conjugated anti-CD15 antibody. For the detection of intracellular virus Nucleoprotein, cells were fixed with 4% paraformaldehyde, permeabilized and blocked with 0.2%Triton X-100, 5%BSA in advance incubated with FITC-conjugated anti-Influenza A Virus Nucleoprotein antibody. After washing, cells were resuspended in HBSS (Solarbio, Beijing, China). Flow cytometry was performed using FACS Aria II instruments (BDBiosciences, San Jose, CA, USA) and Flow Jo software (Tree Star, Inc. USA) was used for data analysis.

### Cell co-culture

The inner surface of 1.0 μm twelve-well millicell (Millipore, Billerica, MA, USA) inserts was coated with fibronectin (Sigma, St. Louis, MO, USA) to ensure neutrophil attachment. Human neutrophils were exposed to influenza virus in twelve-well millicell inserts at 37 °C for 1 h to allow viral infection. Then the culture supernatant was discarded and cells were washed with HBSS (Solarbio, Beijing, China) three times to make sure unbound viruses were removed. The inserts with virus-exposed neutrophils were put into twelve-well plates with cultured human colorectal mucosa cell line (FHC). Cells were collected at designated durations post infection and were analyzed with immunocytochemistry and flow cytometry. Immunocytochemistry was performed as described previously^55^ with rabbit anti-Influenza A Virus Nucleoprotein as primary antibody (Gene Tex, Irvine, CA, USA). Flow cytometry was performed as described above.

### MTS assay

Separated neutrophils were plated in a 96-well plate and then infected with influenza virus. A time course experiment was performed to monitor cell viability with a Cell Titer 96 AQueous One Solution Cell Proliferation Assay Kit (Promega, Madison, WI, USA) according to the manufacturer’s instructions. Uninfected neutrophils were examined in parallel as control.

### Cell apoptosis analysis

At 4 h, 8 h, 12 h and 24 h post infection, neutrophils were collected and analyzed with a Annexin V-FITC/PI Apoptosis Detection Kit (Beyotime, Jiangshu, China) following the manufacturer’s instructions. Flow cytometry was performed using FACS Aria II instruments (BDBiosciences, San Jose, CA, USA) and Flow Jo software (Tree Star, Inc. USA) was used for data analysis. The same time points of uninfected neutrophils were examined as control.

### Neutrophil activation measurement

H2O2 production was measured with a Hydrogen Peroxide Assay Kit (Beyotime, Jiangshu, China) following the manufacturer’s instructions. Activation was measured by incubating neutrophils with influenza virus for 30 min or 10–7 M N-formyl-L-methionyl-L-leucyl-L-phenylalanine (FMLP, Sigma, St. Louis, MO, USA) for 5 min. Deactivation was assessed by first incubating neutrophils with influenza virus for 30 min followed by activated with 10–7 M FMLP for 5 min. Neutrophils without any treatment was used as negative control.

### Statistical Analysis

Statistical analyses were performed using Prism (GraphPad Software, La Jolla, CA, USA). All experiments were repeated in neutrophils derived from at least three different individuals. The data were expressed as the mean ± S.D. and compared with Student’s t-test or One-way ANOVA. Comparisons of each group were performed with q-test (Newman-Keul’s test). The significant level was set at P < 0.05.

## Additional Information

**How to cite this article**: Zhang, Z. *et al.* Infectious Progeny of 2009 A (H1N1) Influenza Virus Replicated in and Released from Human Neutrophils. *Sci. Rep.* **5**, 17809; doi: 10.1038/srep17809 (2015).

## Supplementary Material

Supplementary Information
