# Supplementary material for: Infectious Progeny of 2009 A (H1N1) Influenza Virus Replicated in and Released from Human Neutrophils
Source: Sci Rep. 2015 Dec 7;5:17809. doi: 10.1038/srep17809 (PMC4671072; doi:10.1038/srep17809)
Supplement: Supplementary Information [file srep17809-s1.pdf]

Infectious Progeny of 2009 A (H1N1) Influenza Virus Replicated in and Released from Human Neutrophils

Zhang Zhang[1,9], Tao Huang[1,9], Feiyuan Yu[1], Xingmu Liu[1], Conghui Zhao[2], Xueling Chen[1], David J. Kelvin[3,4,5,6,7], Jiang Gu[1,2,8, *]

1. Department of Pathology and Provincial Key Laboratory of Infectious Diseases and Immunopathology, Collaborative and Creative Center, Shantou University Medical College, Shantou, Guangdong, 515041, China
2. Department of Pathology, Beijing University Health Science Center, Beijing, 100083, China
3. Division of Experimental Therapeutics, Toronto General Research Institute, University Health Network, Toronto, Ontario, Canada
4. Department of Immunology, Faculty of Medicine, University of Toronto, Toronto, Ontario, Canada
5. Universita' degli Studi di Sassari, Sezione di Microbiologia Sperimentale e Clinica, Dipartimento di Scienze Biomediche, Viale San Pietro 43/b, 07100 Sassari, Italia
6. International Institute of Infection and Immunity, Shantou University Medical College, Shantou, Guangdong, China
7. Institute of Medical Science, Faculty of Medicine, University of Toronto, Toronto, Ontario, Canada
8. Translational Medicine Center, Second Affiliated Hospital, Shantou University Medical College, Shantou, China

9. These authors contributed equally to this work.

*. Corresponding author: Jiang Gu, MD, PhD

Supplementary Figure.

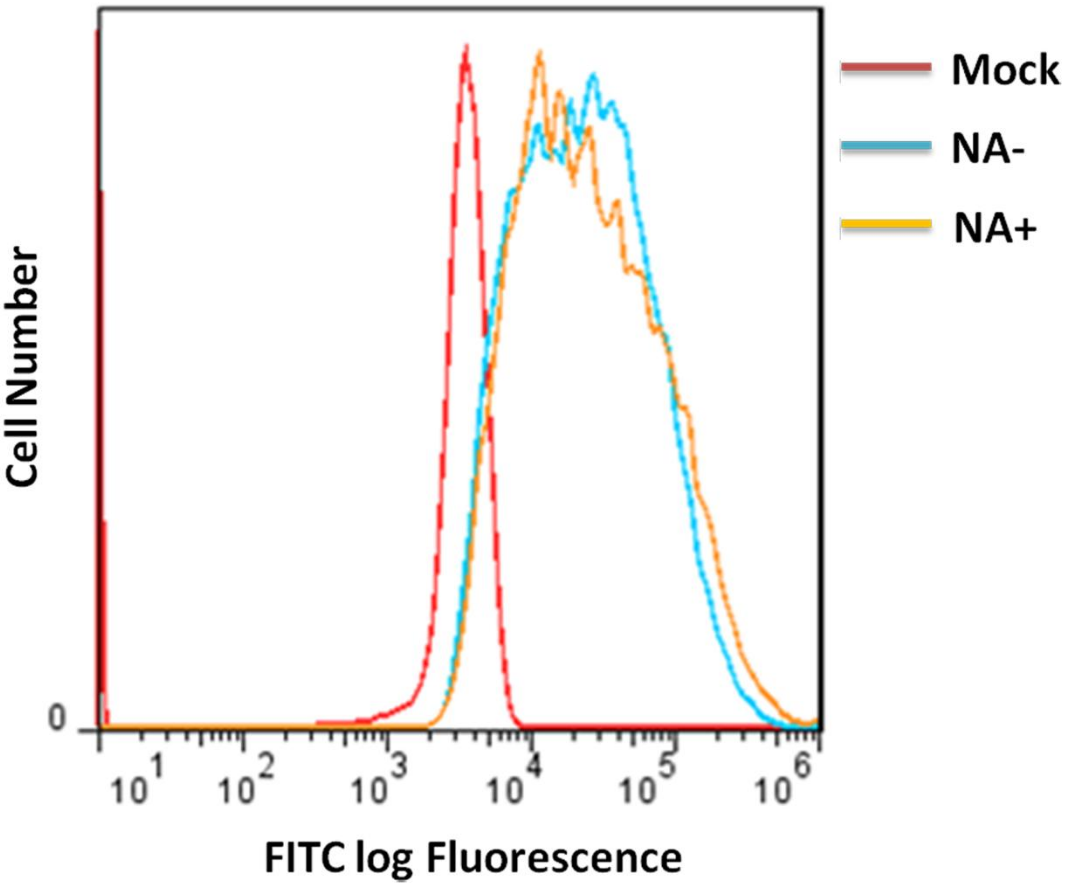

Figure S1. Neuraminidase (NA) pretreatment of neutrophils did not reduce the percentage of infected neutrophils.

Neutrophils were preincubated with NA prior to infection with influenza virus in order to remove the possible existence of sialic acid residues. Cells were infected (MOI 1) with 2009 A (H1N1) influenza virus followed by staining with FITC-labled mouse anti-Nucleoprotein antibody, and then examined with flow cytometry. The Nucleoprotein positive cells represent infected neutrophils. The percentage of neutrophils pretreated with NA (NA+) had nucleoprotein positive cells (infected cells)

in number not less than that of the group of neutrophils without NA treatment (NA-).

Uninfected neutrophils (Mock) were used as negative control.
